# Synthesis and Structure-Activity Relationships of Fenbufen Amide Analogs

**DOI:** 10.3390/molecules15128796

**Published:** 2010-12-02

**Authors:** Kun-I Lin, Chao-Hsun Yang, Chia-Wen Huang, Jhen-Yi Jian, Yu-Chun Huang, Chung-Shan Yu

**Affiliations:** 1 Department of Biomedical Engineering and Environmental Sciences, National Tsing-Hua University, Hsinchu 30013, Taiwan; E-Mails: konyi@xuite.net (K.-I.L); chia-wen0827@yahoo.com.tw (C.-W.H).; 2 Department of Obstetrics & Gynecology, Chang Bing Show Chwan Memorial Hospital, Lukang Zhen, Changhua County, Taiwan; 3 Department of Cosmetic Science, Providence University, Taichung 43301, Taiwan; E-Mails: chyang@pu.edu.tw (C.-H.Y.); lfil6169@gmail.com (J.-Y.J).

**Keywords:** macrophage RAW 264.7, nitric oxide, anti-inflammatory, fenbufen amide analog

## Abstract

The previous discoveries of butyl fenbufen amide analogs with antitumor effects were further examined. The amide analogs with 1, 3, 4 and 8 carbons chains were prepared in 70–80% yield. Fenbufen had no cytotoxic effects at concentrations ranging from 10 to 100 μM. Methyl fenbufen amide had significant cytotoxic effects at a concentration of 100 μM. As the length of the alkyl amide side chain increased, the cytotoxic effects increased, and the octyl fenbufen amide had the greatest cytotoxic effect. After treatment with 30 μM octyl fenbufen amide, nearly seventy percent of the cells lost their viability. At the concentration of 10 μM, fenbufen amide analogs did not show cytotoxicity according to the MTT assay results. The NO scavenging activities of the fenbufen amide analogs were not significantly different from those of fenbufen.

## 1. Introduction

The library and *in-situ* enzymatic screening protocol was firstly developed by Wong *et al.* [1,2,3,4]. Our work has successfully extended this concept to *in-situ* screening at a cellular level. The previous study on the library and *in-situ* screening indicated that fenbufen, a non-steroidal anti inflammatory drug, could be modified with a butyl group through an amide formation reaction [5]. The derived butyl amide analogs of fenbufen were found to display significant anti-tumor effects. These findings encouraged a further study of the relationship between chemical structure and bioactivity. For example, whether the effect was mediated through cycloxygenase, a transmembrane protein responsible for inflammatory signaling. In this paper we describe our attempts to synthesize alkyl substituted fenbufen analogs with 1, 3, 4 and 8 carbon chains and the evaluation of their cell toxicities and NO suppression effects on RAW 264.7 cells.

Anti-inflammatory compounds have been investigated in many studies for their potential *in vitro* inhibitory effects using lipopolysaccharide (LPS)-stimulated macrophages [6]. In this system, bacterial LPS is one of the best-characterized stimuli used to induce upregulation of pro-inflammatory proteins such as cyclooxygenase-2 (COX-2) and inducible nitric oxide synthase (iNOS) [7]. Inducible COX-2 is responsible for the high prostaglandin levels observed in many inflammatory pathologies [8]. Similarly, iNOS produces large amounts of nitric oxide (NO) and is thought to play a central role in inflammatory disease [9]. Numerous studies have reported that NO and prostaglandin (PGE_2_) participate in inflammatory and nociceptive events [10].

## 2. Results and Discussion

### 2.1. Synthesis of the fenbufen amide analogs

**Figure 1:**
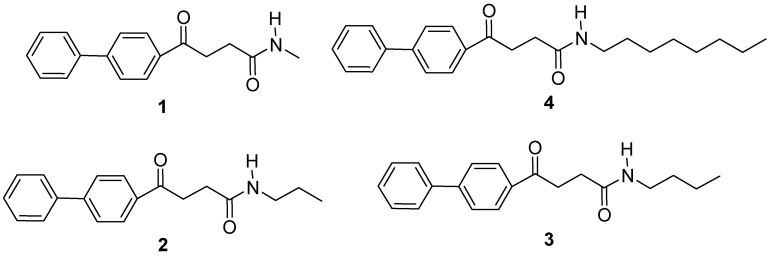
Fenbufen amide analogs: methyl fenbufen (**1**); propyl fenbufen (**2**); butyl fenbufen (**3**); octyl fenbufen (**4**).

Preparation of the fenbufen amide analogs **1-4** (Figure 1) was accomplished in a good yield (70-80%). according to the usual coupling condition as described before. A chromatographic purification on silica gel was employed and spectroscopic data, including ^1^H-NMR and ESI-MS, were consistent with the structures and fully confirmed the identity of these analogs. 

### 2.2. Effects of fenbufen amide analogs on cell viability 

**Figure 2:**
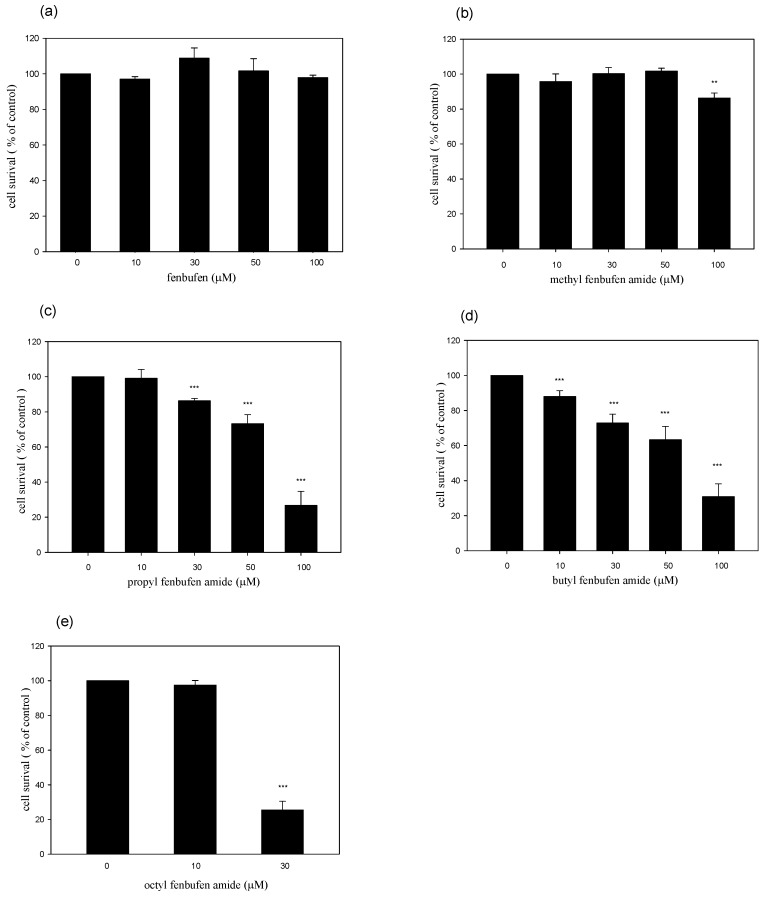
Effects of fenbufen and its amide analogs on cell viabilities in RAW 264.7 cells. Cell viability was estimated using mitochondria MTT assay: **(a)** fenbufen; **(b)** methyl fenbufen amide; **(c)** propyl fenbufen amide; **(d)** butyl fenbufen amide; **(e)** octyl fenbufen amide. *** *p* < 0.001 indicate statistically significant differences.

As a first step towards determining the effects of fenbufen derivatives on NO production, we measured the cell number in RAW 264.7 cells. Cells treated with various concentrations (10-100 μM) of the fenbufen amide analogs were estimated using the mitochondria MTT reduction assay. These results demonstrated that fenbufen had no cytotoxic effect at concentrations ranging from 10 to 100 μM (Figure 2a). According to the results from Figure 2, we found that the methyl fenbufen amide had the significant cytotoxic effect at the concentrations of 100 μM. As the length of the alkyl substituted chain increased, the cytotoxic effects increased, and the octyl fenbufen amide analogue had the greatest cytotoxic effect. After treatment with 30 μM octyl fenbufen amide, nearly seventy percent of the cells lost their viability (Figure 2b–e).

### 2.3. Effects of fenbufen amide analogs on NO production in LPS-activated RAW 264.7 cells

**Figure 3:**
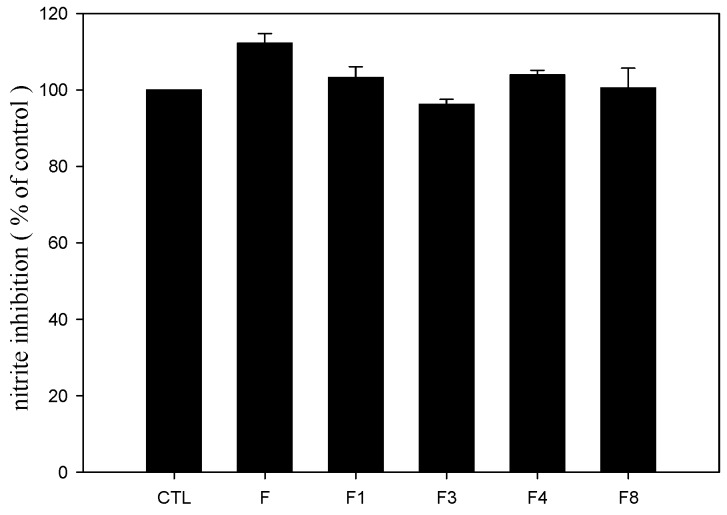
Effect of fenbufen amide analogs on LPS-activated NO production in RAW 264.7 cells. Nitrite was measured using Griess reaction at 24 h after treatment with LPS (100 ng/ml) in the presence or absence fenbufen and its amide analogs (10 μM). All data were presented as the mean ± S.D. of four independent experiments. CTL, control; F, fenbufen; F1, methyl fenbufen amide; F2, ethyl fenbufen amide; F3, propyl fenbufen amide; F8, octyl fenbufen amide.

NO in LPS-activated RAW 264.7 cells was measured by the accumulation of nitrite, the stable metabolite of NO, in the culture broth. At the concentration of 10 μM used in the study, the fenbufen amide analogs did not show cytotoxicity according to the MTT assay results. The NO scavenging activities of the fenbufen amide analogs were not significant different from that of fenbufen (Figure 3).

## 3. Experimental 

### 3.1. General 

DMF was dried and distilled over CaH_2_. The distillate was collected and stored over 4 Å MS until use. The eluents for chromatography, including EtOAc, acetone, and *n*-hexane, were industrial grade and distilled before use. CHCl_3_ was reagent grade and used without further purification. NMR spectroscopy including ^1^H-NMR (500 MHz) and ^13^C-NMR (125 MHz, DEPT-135) was performed either at the Department of Chemistry of National Tsing-Hua University (NTHU) or the Department of Applied Chemistry of National Chiao-Tung University (NCTU), both employing Varin Unity Inova 500NMR. Deuterated solvents employed for NMR spectroscopy including CD_3_OD, CDCl_3_ and C_6_D_6_ were purchased from Cambridge Isotope Laboratories, Inc. ESI-MS spectrometry employing a Micromass Q-Tof liquid chromatography tandem mass spectrometer was performed at the Department of Applied Chemistry of National Chiao-Tung University (NCTU). TLC was performed with Machery-Nagel silica gel 60 F254 precoated plates. The starting materials and products were visualized by UV (254 nm). Further confirmation was carried out by using staining with 5% *p*-anisaldehyde, ninhydrin or ceric ammonium molybdate [Ce(NH_4_)_2_(NO_3_)_6_, (NH_4_)_6_Mo_7_O_24_·H_2_O] under heating. Flash chromatography was performed using Geduran Si 60 silica gel (230-400 mesh).

### 3.2. Typical procedure for the coupling 

The chemical synthesis for the amide products followed a similar procedure to that described before [3].

*N-methyl-4-(biphenyl-4-yl)-4-oxobutanamide* (**1**). Anal. C_17_H_17_NO_2_, M (calcd.) = 267.2 (m/z), ESI+Q-TOF: M = 267.2 (m/z), [M+H]^+^ = 268.2 (99%), 269.2 (18%), [M+Na]^+^ = 290.2 (100%), 291.2 (15%), [2M+H]^+^ = 535.3 (6%), [2M+Na]^+^ = 557.2 (53%), 558.2 (19%), 559.3 (3%), equivalent to the calculated isotopic ratio 100:38:8 for [2M+Na]^+^; ^1^H-NMR (CD_6_D_6_): δ 1.11 (t, 3H, CH_3_), 2.26 (t, 2H, *J* = 6.5 Hz, 2H, CH_2_), 3.08 (t, 2H, *J* = 6.5 Hz, 2H, CH_2_), 4.52 (bs, 1H, CONH,), 7.16-7.22 (m, 3H, aromatic), 7.33-7.37 (m, 4H, aromatic), 7.89-7.92 (m, 2H, aromatic).

*N-propyl-4-(biphenyl-4-yl)-4-oxobutanamide* (**2**). Anal. C_19_H_21_NO_2_, M (calcd.) = 295.2 (m/z), ESI+Q-TOF: M = 295.2 (m/z), [M+H]^+^ = 296.3 (100%), 269.2 (19%), [M+Na]^+^ = 318.2 (74%), 319.2 (10%), [2M+H]^+^ = 591.3 (3%), [2M+Na]^+^ = 613.3 (67%), 614.3 (28%), 615.3 (6%), equivalent to the calculated isotopic ratio 100:42:9 for [2M+Na]^+^; ^1^H-NMR (CD_3_OD): δ 0.93 (t, *J* = 7.0 Hz, 3H, CH_3_), 1.52 (sextet, *J* = 6.5 Hz, 2H, CH_2_), 2.61 (t, 2H, *J* = 7.0 Hz, 2H, CH_2_), 3.14 (t, *J* = 7.0 Hz, 2H, CH_2_), 3.36 (t, *J* = 6.5 Hz, 2H, CH_2_), 7.37-7.40 (m, 1H, aromatic), 7.45-7.48 (m, 2H, aromatic), 7.66-7.68 (m, 2H, aromatic), 7.74-7.75 (m, 2H, aromatic), 8.06-8.08 (m, 2H, aromatic).

*N-butyl-4-(biphenyl-4-yl)-4-oxobutanamide* (**3**). A solution of fenbufen (214 mg, 0.8 mmol), HBTU (302 mg, 0.8 mmol), DIEA (1 mL, 0.8 mmol) and DMF (15 mL) was stirred. The corresponding carboxylic acid analog was added. TLC (acetone/*n*-hexane = 3/7) indicated the consumption of starting material (R*_f_* = 0.39) and the formation of the product (R*_f_* = 0.78). After stirring for 1 h, the mixture was concentrated under high vacuum with oil pump at 60 °C. The residue was purified by flash chromatography using acetone/*n*-hexane 3:7 as eluents to provide a snow-white solid in 77% yield (269 mg). Anal. C_20_H_23_NO_2_, M (calcd.) = 309.2 (m/z), ESI+Q-TOF: M = 309.2 (m/z), [M+H]^+^ = 310.3 (68%), 311.3 (13%), [M+Na]^+^ = 332.2 (65%), 333.2 (12%), [2M+Na]^+^ = 641.3 (100%), 642.3 (42%), 643.3 (10%), equivalent to the calculated isotopic ratio 100:44:10 for [2M+Na]^+^; ^1^H-NMR (CD_3_OD): δ 0.78 (t, 3H, CH_3_, *n*-butyl), 1.28-1.40 (m, 2H, CH_2_, *n*-butyl), 1.46-1.52 (m, 2H, CH_2_, *n*-butyl), 2.60 (t, 2H, *J* = 6.5 Hz, 2H, CH_2_), 3.18 (t, 2H, *J* = 7.0 Hz, 2H, CH_2_), 3.35 (t, 2H, *J* = 6.5 Hz, 2H, CH_2_), 7.37-7.42 (m, 1H, aromatic), 7.46-7.48 (m, 2H, aromatic), 7.67-7.68 (m, 2H, aromatic), 7.74-7.76 (m, 2H, aromatic), 8.07-8.08 (m, 2H, aromatic); ^1^H-NMR (C_6_D_6_): δ 0.78 (t, 3H, CH_3_, *n*-butyl), 1.07-1.16 (m, 2H, CH_2_, *n*-butyl), 1.16-1.23 (m, 2H, CH_2_, *n*-butyl), 2.33 (dd, 2H, *J* = 6.5 Hz, 2H, CH_2_), 3.07 (ddd, *J* = 7.0 Hz, 2H, CH_2_, [(CONH)CH_2_, *n*-butyl], 3.11 (dd, *J =* 7.0 Hz, 2H, HNCOCH_2_), 4.83 (bs, 1H, CONH), 7.10-7.22 (m, 3H, aromatic), 7.32-7.39 (m, 4H, aromatic), 7.88-7.93 (m, 2H, aromatic); ^13^C-NMR (C_6_D_6_): δ 13.9 (CH_3_), 20.2 (CH_2_), 30.4 (CH_2_), 32.0 (CH_2_), 34.3 (CH_2_), 39.3 (CH_2_), 127.4 (CH), 127.5 (CH), 128.2 (CH), 129.0 (CH), 129.1 (CH), 136.0 (C), 140.4 (C), 145.7 (C), 171.1 (C), 198.1 (C).

*N-octyl-4-(biphenyl-4-yl)-4-oxobutanamide* (**4**). Anal. C_24_H_31_NO_2_, M (calcd.) = 365.2 (m/z), ESI+Q-TOF: M = 365.3 (m/z), [M+H]^+^ = 366.3 (30%), 367.3 (7%), [M+Na]^+^ = 388.3 (15%), 389.3 (4%), [2M+H]^+^ = 731.5 (8%), 732.5 (4%), [2M+Na]^+^ = 753.4 (13%),754.4 (8%), equivalent to the calculated isotopic ratio 100:26 for [M+H]^+^; ^1^H-NMR (C_6_D_6_): δ 0.89 (t, *J* = 7.0 Hz, 3H, CH_3_), 1.15-1.22 (m, 8H, CH_2_), 1.25-1.30 (m, 4H, CH_2_), 2.36 (t, *J* = 7.0 Hz, 2H, CH_2_), 3.10-3.14 (m, 4H, CH_2_), 7.10-7.22 (m, 3H, aromatic), 7.33-7.38 (m, 4H, aromatic), 7.90-7.92 (m, 2H, aromatic).

### 3.3. Biological assay chemicals and reagents 

Fetal bovine serum, dimethyl sulfoxide, lipopolysaccharide (LPS, *Escherichia coli* serotype 055:B5), sulfanilamide, *N*-(1-naphthyl)ethylenediamine, phosphoric acid, sodium nitrite, α,α-diphenyl-2-picrylhydrazyl, 3-[4,5-dimethylthiazol-2-yl]-2,5-diphenyltetrazolium bromide (MTT), and fenbufen were purchased from Sigma-Aldrich Chemical Co. (St. Louis, MO, USA). All samples were dissolved in dimethylsulfoxide (DMSO) and further diluted in culture medium. The final DMSO concentration in the medium was 0.1% and did not affect cellular function or the assay systems used in this study.

### 3.4. Cell culture 

The murine macrophage cell line RAW 264.7 was purchased from Bioresource Collection and Research Center (BCRC, Taiwan). RAW264.7 cells were cultured in Dulbecco’s modified Eagle’s medium (DMEM) supplemented with antibiotics (100 U/mL of penicillin, 100 μg/mL of streptomycin, and 0.25 μg/mL of amphotericin B), and 10% heat-inactivated fetal bovine serum (FBS). The cells were incubated in a humidified incubator at 37 °C in 5% CO_2_/air.

### 3.5. Cell viability assay 

Cell viability was determined using MTT assay [11]. Briefly, cells were seeded at a density of 7 × 10^4^/mL on 96-well plates and cultured overnight as described above. The medium was then replaced with fresh medium containing fenbufen derivatives at various concentrations. After incubation for 24 h at 37 °C in 5% CO_2_/air, MTT (final concentration, 0.5 mg/mL) was added, and the cells were then incubated at 37 °C for 2 h. Finally, the cells were lysed and absorbance was detected at 550 nm. For cell number determination, a standard correlation between the known cell numbers and the absorbance density values was constructed for measuring the cell number from various detected absorbance density values.

### 3.6. Determination of NO production 

In order to measure NO production, macrophage RAW 264.7 cells were plated into 96-well plate (5 × 10^5^ cell/mL) and treated with 100 ng/mL of LPS in the presence or absence of fenbufen derivatives for 24 h. Nitrite, a soluble oxidation product of NO, in the culture supernatanr, was determined using the Griess reaction [12]. The supernatant (50 μL) was harvested and mixed with an equal volume of Griess reagent (1% sulfanilamide and 0.1% naphthylethylenediamine dihydrochloride in 5% phosphoric acid). After 10 min, the absorbance at 540 nm was measured using a microplate reader. Sodium nitrite was used as a standard to calculate NO_2_^-^ concentration.

### 3.7. Statistical analysis 

Data are expressed as mean ± S.D. of the indicated number of separate experiments. A one-way analysis of variance was performed for multiple comparisons, and if there was significant variation between treatment groups, the mean values were compared with the respective control using Student’s *t*-test. *P* values less than 0.05 were considered significant.

## 4. Conclusions

The methyl, propyl, butyl and octyl analogs of fenbufen were successfully synthesized in good yield. As the length of the alkyl amide side chain increased, the cytotoxic effects increased. The NO scavenging activities of the fenbufen amide analogs were not significantly different from those of fenbufen itself.

## References

[1] Brik A., Wu C.Y., Wong C.H. (2006). Microtiter plate based chemistry and *in situ* screening: a useful approach for rapid inhibitor discovery. Org. Biomol. Chem..

[2] Best M., Brik A., Chapman E., Lee L., Cheng W.C., Wong C.H. (2004). Rapid discovery of potent sulfotransferase inhibitors by diversity-oriented reaction in microplates followed by *in situ* screening. Chem. Biol. Chem..

[3] Wu C.Y., Chang C.F., Chen J.S.Y., Lee S.T., Wong C.H., Lin C.H. (2003). Rapid diversity-oriented synthesis in microtiter plates for *in situ* screening: discovery of potent and selective fucosidase inhibitors. Angew. Chem. Int. Ed..

[4] Brik A., Lin Y.C., Elder J., Wong C.H. (2002). A quick diversity-oriented amide-forming reaction to optimize P-subsite residues of HIV protease inhibitors. Chem. Biol..

[5] Chiang L.W., Pei K., Chen S.W., Huang H.L., Lin K.J., Yen T.C., Yu C.S. (2009). Combining a solution-phase derived library with in-situ cellular bioassay: Prompt screening of amide-forming minilibraries using MTT assay. Chem. Pharm. Bull..

[6] Yang E.J., Moon J.Y., Kim M.J., Kim D.S., Kim C.S., Lee W.J., Lee N.H., Hyun C.G. (2010). Inhibitory effect of *Jeju endemic* seaweeds on the production of pro-inflammatory mediators in mouse macrophage cell line RAW 264.7. J. Zhejiang Univ. Sci. B..

[7] Jean Y.H., Chen W.F., Duh C.Y., Huang S.Y., Hsu C.H., Lin C.S., Sung C.S., Chen I.M., Wen Z.H. (2008). Inducible nitric oxide synthase and cyclooxygenase-2 participate in anti-inflammatory and analgesic effects of the natural marine compound lemnalol from Formosan soft coral *Lemnalia cervicorni*. Eur. J. Pharmacol..

[8] Yoon W.J., Ham Y.M., Yoo B.S., Moon J.Y., Koh J., Hyun C.G. (2009). *Oenothera laciniata* inhibits lipopolysaccharide induced production of nitric oxide, prostaglandin E_2_, and proinflammatory cytokines in RAW264.7 macrophages. J. Biosci. Bioeng..

[9] Gaspirc B., Masera A., Skaleric U. (2002). Immunolocalization of inducible nitric oxide synthase in localized juvenile periodontitis patients. Connect. Tissue Res..

[10] Yoon W.J., Kim S.S., Oh T.H., Lee N.H., Hyun C.G. (2009). *Abies koreana* essential oil inhibits drug-resistant skin pathogen growth and LPS-induced inflammatory effects of murine macrophage. Lipids.

[11] Wang G.J., Chen Y.M., Wang T.M., Lee C.K., Chen K.J., Lee T.H. (2008). Flavonoids with iNOS inhibitory activity from *Pogonatherum crinitum*. J. Ethnopharmacol..

[12] Chiang L.W., Pei K., Chen S.W., Huang H.L., Lin K.J., Yen T.C., Yu C.S. (2009). Combining a solution-phase derived library with in-situ cellular bioassay: Prompt screening of amide-forming minilibraries using MTT assay. Chem. Pharm. Bull..

